# Evaluation of a near-infrared version of TMR-PEG1k, a high-performance untargeted contrast agent for fluorescence-guided surgery, using fluorescence cryotomography

**DOI:** 10.1117/1.JBO.30.12.126004

**Published:** 2025-12-04

**Authors:** Augustino V. Scorzo, Caleb Y. Kwon, Rendall R. Strawbridge, David W. Roberts, Keith D. Paulsen, Scott C. Davis

**Affiliations:** a Thayer School of Engineering, Dartmouth College, Hanover, New Hampshire, United States; b Geisel School of Medicine, Dartmouth College, Hanover, New Hampshire, United States; c Dartmouth Cancer Center, Dartmouth-Hitchcock Medical Center, Lebanon, New Hampshire, United States

**Keywords:** contrast agents, fluorescence-guided surgery, near-infrared imaging, fluorescence cryotomography

## Abstract

**Significance:**

In previous studies, we reported on a new untargeted contrast agent, TMR-PEG1k, that exhibits high and durable diagnostic performance in glioma models starting within minutes of administration. This agent uses a fluorophore in the visible regime, which, although it helps ensure high-resolution imaging of superficial tissue, precludes the detection of subsurface structures due to the limited optical penetration. Thus, development of a near-infrared (NIR) version of the agent that retains the properties of TMR-PEG1k would combine the favorable diagnostic performance of TMR-PEG1k with sensitivity to subsurface pathologies enabled by NIR imaging.

**Aim:**

We aim to determine whether exchanging the fluorophore on TMR-PEG1k from tetramethylrhodamine to cyanine 7 would retain the highly favorable imaging properties exhibited by TMR-PEG1k.

**Approach:**

Mice (N=5) with orthotopic gliomas expressing green fluorescent protein (GFP) were co-administered TMR-PEG1k and Cy7-PEG1k. After 30 min, animals were euthanized, and the whole-body specimens were imaged using fluorescence cryotomography to recover co-registered three-dimensional (3D) fluorescence distributions of all fluorescent reporters. We evaluated the two agents using tumor-to-background ratio (TBR), contrast-to-noise ratio (CNR), area under the receiver operating characteristic curve (ROC-AUC), and normalized cross-correlation with GFP fluorescence (CC to GFP).

**Results:**

Although the imaging system exhibited higher sensitivity to Cy7-PEG1k in phantoms, the 3D cryotomography results showed dramatic differences in the properties of the two agents *in vivo*. TMR-PEG1k produced highly selective tumor enhancement and concordance with GFP, whereas Cy7-PEG1k showed much lower selectivity, lower signal intensity, and produced no enhancement in many tumor regions. These observations were confirmed by the evaluation metrics, which were found to be (1) TBR = 5.2 versus 1.7 (p=0.0037), (2) CNR = 17.7 versus 3.8 (p=0.046), (3) ROC-AUC = 0.999 versus 0.821, and (4) CC to GFP = 0.90 versus 0.52, for TMR-PEG1k versus Cy7-PEG1k, respectively.

**Conclusions:**

Cy7-PEG1k did not retain the favorable properties exhibited by TMR-PEG1k and thus is not a suitable NIR analog for this agent.

## 1 Introduction

Fluorescence-guided surgery (FGS) has emerged as a clinical standard for some indications, and the investigation of new agents and imaging strategies is an active area of research and development efforts.^1^^–^^4^ In this approach, fluorescent contrast agents or precursors to endogenous fluorescent compounds are administered to a patient, and a fluorescence imaging device is often used to reveal the contrast agent distribution within the surgical field. The majority of FGS contrast agents, either approved by the Food and Drug Administration or currently under development, are used to delineate tumors from normal tissues during cancer surgeries.^5^ This tumor contrast is typically realized by either passive accumulation of contrast agent within the tumor region or by active selectivity of the agent using a targeting moiety to overexpressed molecules, receptors, or enzymes.

Recently, we reported that the administration of TMR-PEG1k, a nontargeted agent that consists of the fluorophore tetramethylrhodamine (TMR) conjugated to a 1-kDa mPEG chain, produced favorable characteristics for the surgical guidance of glioma.^6^ Specifically, in preclinical orthotopic brain tumor models, this agent produced (1) high tumor-to-normal brain contrast, (2) high diagnostic performance, and (3) high correlation to contrast-enhanced MRI, all within minutes of administration. Moreover, the behavior was durable over 90 min (the longest time measured). Although these encouraging results have prompted further development and evaluation of this agent in large animal models, the fluorophore used for this agent operates in the visible regime (400 to 700 nm), which generally provides sharp, high-resolution images yet has limited sensitivity to subsurface structures. Although some common contrast agents, such as fluorescein sodium and ALA-induced protoporphyrin IX, fluoresce in the visible waveband,^7^^–^^9^ the bulk of FGS contrast agents currently under development operate in the near-infrared (NIR) regime (fluorescence emission >700  nm)^10^^–^^16^ to increase sensitivity to deeper objects and minimize tissue autofluorescence.^12^^,^^13^^,^^15^ In this context, the development of an NIR version of TMR-PEG1k would combine the highly favorable kinetic behavior observed previously with the advantages of subsurface sensitivity.

In this study, we directly compare the three-dimensional (3D) fluorescence distributions of TMR-PEG1k with an NIR analog, Cy7-PEG1k, the latter of which consists of cyanine 7 conjugated to a 1-kDa mPEG chain, in preclinical models. Specifically, we used fluorescence cryotomography of mice containing green fluorescent protein (GFP)-expressing gliomas co-administered with both untargeted contrast agents. The multichannel fluorescence cryotomography acquisition provided high-resolution, co-registered 3D fluorescence image volumes of ground-truth GFP and of each agent. Contrast agent performance was evaluated using tumor-to-background ratio (TBR), contrast-to-noise ratio (CNR), area under the receiver operating characteristic curve (ROC-AUC), and normalized cross-correlation to the GFP fluorescence volume (CC to GFP).

## 2 Materials and Methods

### 2.1 Experimental Design

This study was designed to evaluate and compare a near-infrared analog (Cy7-PEG1k) to TMR-PEG1k. TMR-PEG1k is a new contrast agent that has shown high, rapid, and persistent diagnostic performance as well as similar to contrast-enhanced MRI in preclinical brain tumor models.^6^ Mice (N=5) with orthotopic brain tumors expressing GFP were co-administered Cy7-PEG1k and TMR-PEG1k in the tail vein and euthanized 30 min later. The choice of this time was based on the aim of advancing an agent that produces high performance shortly after administration, the previously reported high diagnostic performance of TMR-PEG1k at this time point, and guidance from the results of a preliminary study examining the kinetics of Cy7-PEG1k in exposed flank tumors in live animals (Fig. S1 in the Supplementary Material). After euthanasia, whole animal specimens were then frozen and imaged using fluorescence cryotomography to produce high-resolution image volumes of RGB, GFP (for ground truth tumor), TMR-PEG1k, and Cy7-PEG1k fluorescence in the same animal. The tumor was segmented using the GFP fluorescence, and the following performance metrics were computed and compared between the two agents: TBR, CNR, ROC-AUC, and CC to GFP. In addition, line intensity profiles of the contrast agents were evaluated using sampled slices within the image volume.

### 2.2 Fluorescent Contrast Agents

Two fluorescent contrast agents were evaluated:

1. TMR-PEG1k: tetramethylrhodamine conjugated to a 1-kDa mPEG chain (Creative PEGWorks, Durham, North Carolina, United States; dosed at 42.1 nmol).
2. Cy7-PEG1k: cyanine 7 conjugated to a 1-kDa mPEG chain (Biopharma PEG, Watertown, MA, United States; dosed at 42.1 nmol).

All contrast agent stocks were prepared from aliquots previously reconstituted in phosphate-buffered saline and kept at −20°C. Before administration, the contrast agent concentration was confirmed using absorption spectrometry.

Normalized absorbance and emission spectra for these agents (shown in Fig. S2 in the Supplementary Material) were recorded using a Cary 50 Bio UV-Visible Spectrophotometer (Varian, Palo Alto, California, United States) and Fluoromax 4 Spectrofluorometer (HORIBA Instruments Incorporated, Irvine, California, United States.), respectively, and the spectra for the free dyes can be found in previous publications.^17^^,^^18^ In addition, physicochemical properties of TMR and Cy7 were computed using MarvinSketch (ChemAxon, Budapest, Hungary) and provided in Fig. S3 in the Supplementary Material.

### 2.3 Phantom Imaging to Determine Contrast Agent Detection Limit

Serial dilution phantoms for each contrast agent were imaged using the fluorescence cryotomography system to determine the minimum detectable concentration for TMR-PEG1k and Cy7-PEG1k. These phantoms consisted of 1% intralipid (Paterson Veterinary Supply, Inc., Greeley, Colorado, United States) diluted in phosphate-buffered saline and dilutions of either TMR-PEG1k or Cy7-PEG1k. For each fluorescence contrast agent, the concentration was serially diluted (1:2) from 200 nM in wells containing 500  μL of solution. The mean fluorescence signal in each well was plotted as a function of true concentration. The lower limit of detection was determined as the lowest concentration that produced a signal greater than three times the standard deviation of the 0-nM background wells as described elsewhere.^19^

### 2.4 Animal Models

All experiments were conducted according to protocols approved by the Institutional Animal Care and Use Committee at Dartmouth College. Female nude mice 7 to 10 weeks old (Charles River Laboratory, Wilmington, Massachusetts, United States) underwent intracranial inoculation with $10^{6}$ U87 GFP-expressing glioma cells (Neuromics, Minneapolis, Minnesota, United States) using a previously described surgical procedure.^20^ Immediately following implantation, animals were placed on a chlorophyll-free diet (MP Biomedicals, Irvine, California, United States) to limit tissue autofluorescence for fluorescence cryotomography. Tumor growth was monitored using contrast-enhanced MRI until the largest observable tumor dimension measured at least 2 mm. Within 4 days of this MRI scan, animals were co-administered the two fluorescent agents in the tail vein, euthanized 30 min later, submerged in optimal cutting temperature solution, and frozen at −20°C in preparation for fluorescence cryotomography.

### 2.5 Whole-Body 3D Fluorescence Cryotomography and Histopathology

The large specimen 3D fluorescence cryotomography system has been described previously.^6^^,^^21^ This technique automatically sections and acquires hyperspectral image stacks of frozen specimens, which are processed into 3D volumes.^6^^,^^22^^–^^29^ In this study, four light sources were used: (1) a white light emitting diode (LED, Mightex, Toronto, Ontario, Canada) for RGB imaging, (2) a 530-nm LED (Mightex, Toronto, Ontario, Canada) with a 542-nm band pass filter (bandwidth of 22 nm) for imaging TMR-PEG1k fluorescence, (3) a 470-nm LED (Mightex, Toronto, Ontario, Canada) with a 475-nm short pass filter for imaging GFP fluorescence, and (4) a 760-nm laser (Custom built Spectra III Lumencor, Beaverton, Oregon, United States) with a 780-nm LP emission filter for imaging Cy7-PEG1k. To limit spectral overlap between TMR-PEG1k and GFP fluorescence, we constrained the analysis of the 530-nm LED excitation channel to the 620- to 720-nm wavebands for TMR-PEG1k emission and 510- to 530-nm wavebands for GFP (470-nm LED channel). The final co-registered image volumes were visualized using 3D Slicer.^30^ All animals in this study were sectioned at 100  μm during the image acquisition process.

### 2.6 Micro-Computed Tomography (CT) Acquisition and Registration

For a subset of the animals, CT scans were acquired of the frozen specimens prior to fluorescence cryotomography using an IVIS SpectrumCT (PerkinElmer, Waltham, Massachusetts, United States) running the Living Image Software. The CT acquisition parameters were CT mode = A50, exposure time = 100 ms, voltage = 50 V, current = 1 A, X-ray filter = 440 Al, and total projections = 720. The reconstructed CT volumes were loaded into 3D Slicer and co-registered to the cryotomography volume using a point-based rigid registration between distinguishable features in the CT and RGB volumes.^30^

### 2.7 Image Analysis and Statistics

The volumetric tumor boundaries were identified using the GFP fluorescence volumes, and the background brain region was chosen as a zone in the contralateral brain of size approximately equal to the whole tumor volume. All animals exhibited multiple regions of tumor growth within the brain, and these regions were combined for the purposes of analysis. These tumor and background regions of interest were then applied to the fluorescence agent volumes, and the following performance metrics were computed as described previously:^6^ TBR, CNR, area under the curve from the receiver operating characteristic analysis (ROC-AUC), and normalized cross-correlation of each fluorescent agent volume to the corresponding GFP volume (CC to GFP). Results were plotted as a median with interquartile ranges. Significant differences between TBR and CNR analyses for the two contrast agents were assessed using a two-tailed paired t-test with the significance level set at 0.05. Statistical analysis was performed using Prism version 10 (GraphPad, San Diego, California, United States).

### 2.8 *In Vitro* Cell Staining

To examine the behavior further of the agents and help elucidate the *in vivo* results, a live cell incubation study was completed. U87 parent line cells were plated and incubated for 30 min in 5  μM of either TMR-PEG1k or Cy7-PEG1k. After incubation, the cells were washed five times with PBS and then imaged on a fluorescence microscope using the following excitation and emission wavebands:

• TMR-PEG1k: excitation = 542 nm, emission filter = 592-nm bandpass.
• Cy7-PEG1k: excitation = 760 nm, emission filter = 780-nm long pass.

## 3 Results

**Fig. 1:**
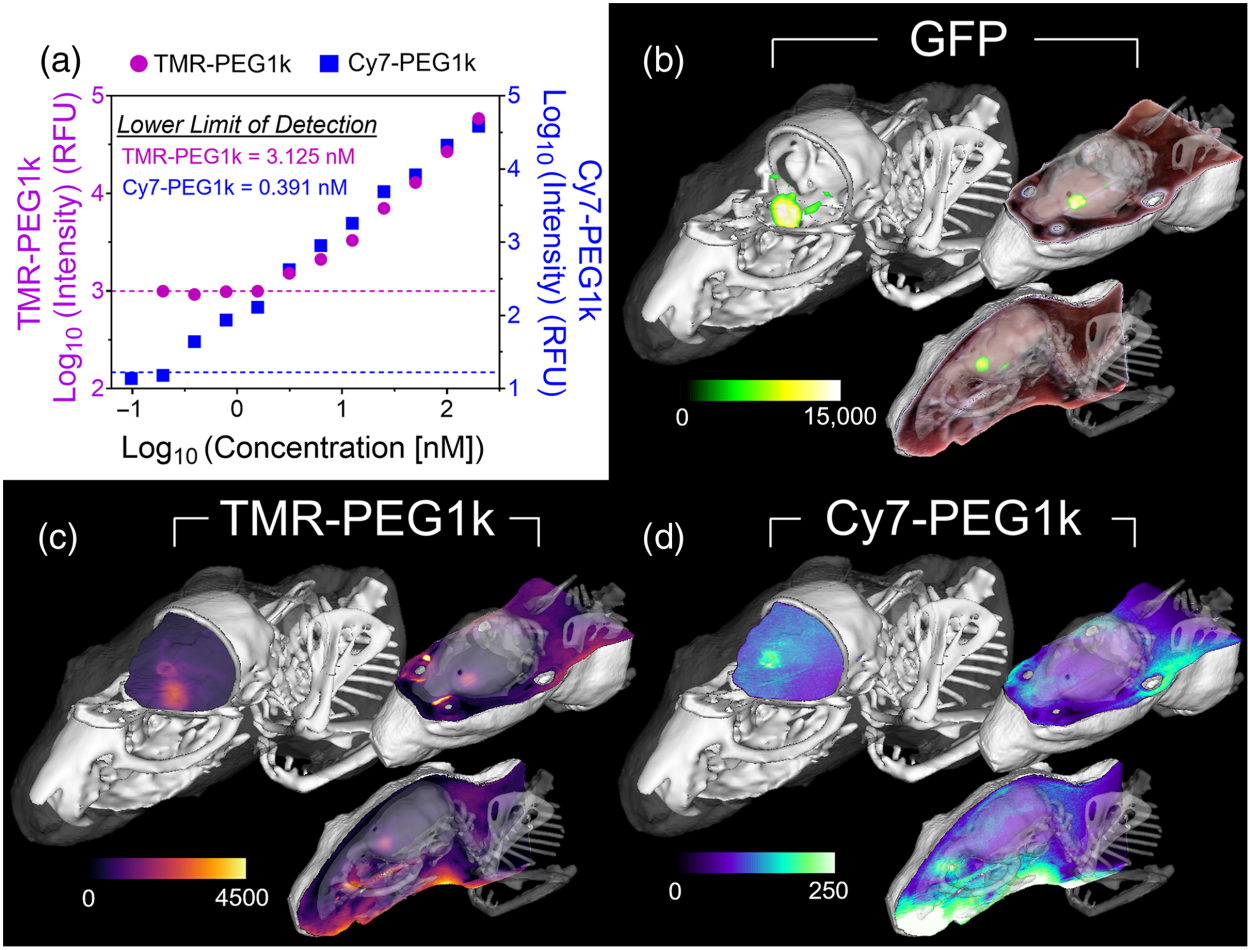
(a) Phantom imaging results of serially diluted fluorescent contrast agent concentrations for TMR-PEG1k and Cy7-PEG1k (lower limit of detections denoted by the dashed lines). (b) Volume renderings of GFP fluorescence. Clockwise from left: GFP fluorescence rendered as an MIP image with CT bone rendering and clipping planes through the skull; coronal RGB image plane with GFP fluorescence overlayed and semitransparent CT bone renderings; sagittal RGB image with GFP overlay and semitransparent CT bone rendering. (c) Clockwise from left: TMR-PEG1k fluorescence rendered as a MIP image with CT bone rendering and clipping planes through the skull; coronal fluorescence image and semitransparent CT bone renderings; sagittal fluorescence image and semitransparent CT bone rendering. (d) Same format as panel (c) but for Cy7-PEG1k. No thresholding was applied to the fluorescence contrast agent volumes.

To quantify the sensitivity of the cryotomography imaging system to the two agents, tissue-simulating phantoms consisting of serial dilutions of each fluorescent agent were imaged. Figure 1(a) shows the fluorescence intensity as a function of the concentration of each agent. These results indicate greater sensitivity to Cy7-PEG1k (lower detection limit = 0.39 nM) than TMR-PEG1k (lower detection limit = 3.13 nM).

Figures 1(b)–1(d) show representative fluorescence cryotomography imaging data (GFP, TMR-PEG1k, and Cy7-PEG1k channels) for one animal. In each panel, the image in the upper left is a maximum intensity projection (MIP) of the fluorescence volume overlaid on a co-registered CT bone volume clipped in two planes. The outer surface of the mouse rendered from the cryotomography data is also depicted. Note that the burr hole used for tumor cell implantation is visible in the CT volumes. Adjacent to the image in the upper left are two additional clipped volume renderings showing a two-dimensional (2D) fluorescence image with a semitransparent rendering of the CT bone volume. Specifically, Fig. 1(b) shows the GFP fluorescence overlaid on RGB images, whereas Figs. 1(c) and 1(d) provide TMR-PEG1k and Cy7-PEG1k volumes, respectively, using the same views as in Fig. 1(b). In the latter two panels, no thresholding was applied to the fluorescence data.

**Fig. 2:**
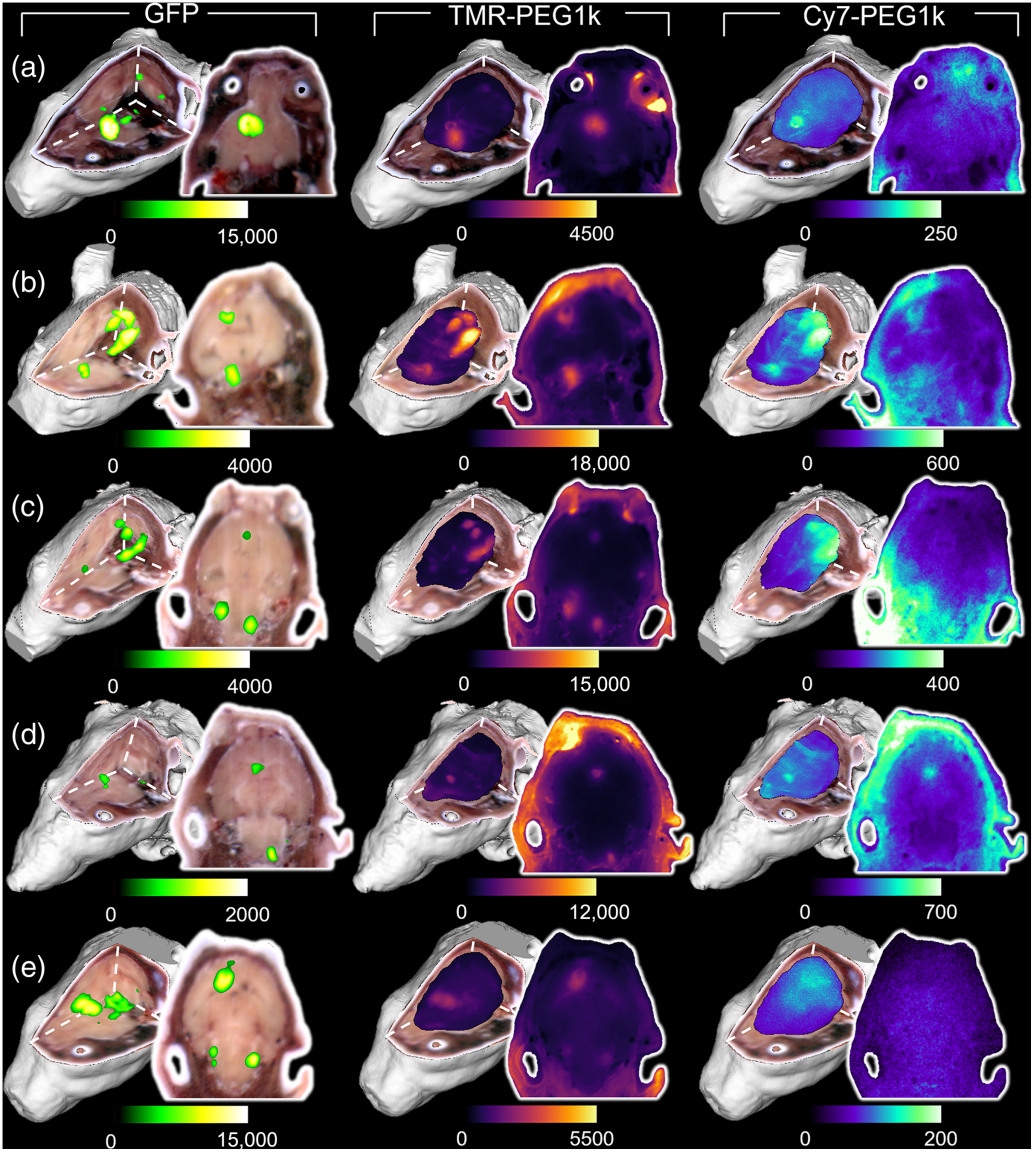
Fluorescence cryotomography volumes of all mice administered contrast agents (a)–(e), with each row showing data for one animal. Columns (from left to right) show GFP, TMR-PEG1k, and Cy7-PEG1k fluorescence renderings. Renderings are displayed as 3D MIPs overlaid on clipped RGB volumes and are accompanied by a select 2D fluorescence image from the volume. Fluorescence volumes are displayed in relative fluorescence units (RFU). A threshold was applied to the GFP images for display, but no threshold was applied to the images of the contrast agents.

**Fig. 3:**
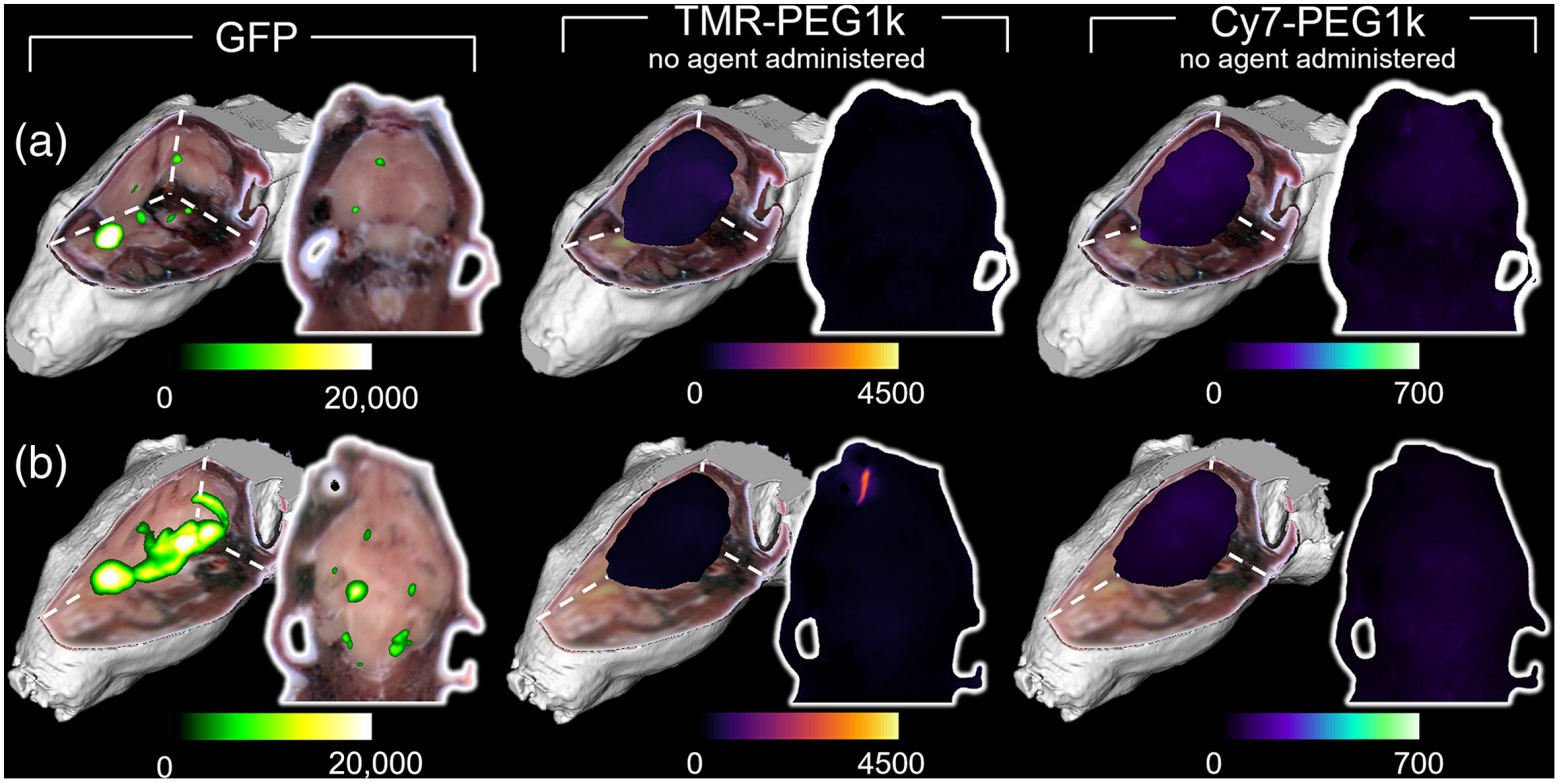
Fluorescence cryotomography volumes of naïve mice without administration of contrast agents (each row represents one animal). Display parameters are identical to those described in Fig. 2.

Fluorescence cryotomography images for all animals administered the fluorescent agents are provided in Fig. 2, and naïve animal results are included in Fig. 3. In both figures, each row depicts data for one animal, whereas the columns are arranged to show GFP, TMR-PEG1k, and Cy7-PEG1k, fluorescence channels, displayed as renderings (MIPs) and a 2D fluorescence image selected from the 3D volumes. A threshold was applied to the GFP volume, but no threshold was applied to the fluorescent contrast agent channels.

Inspection of the GFP fluorescence channel data reveals several tumor regions within each brain, suggesting local metastatic spread of these tumors in all animals. Comparing the contrast agent channels to the GFP channel reveals stark differences between the behavior of the two agents. The TMR-PEG1k channel appears to show high correspondence to the GFP channel data, exhibiting very low normal brain fluorescence and sharp delineation of all tumor regions across animals. This behavior was not observed in the Cy7-PEG1k contrast agent channels, which showed far lower overall signal intensity, poor contrast, and often indistinguishable tumor regions. Evaluating these data alongside the concentration versus RFU curves in Fig. 1(a) indicates that the relative concentration of Cy7-PEG1k was much lower than that of TMR-PEG1k at this time point.

Imaging data for two tumor-bearing control animals provided in Fig. 3 confirm that GFP fluorescence does not contaminate fluorescence emissions received in the other channels. Autofluorescence originating from the harderian gland (not present in humans) around the eye is observed in the TMR-PEG1k imaging channel [Fig. 3(b)]. This feature was also observed in the 2D TMR-PEG1k images of animals depicted in Figs. 2(a), 2(c), and 2(d).

**Fig. 4:**
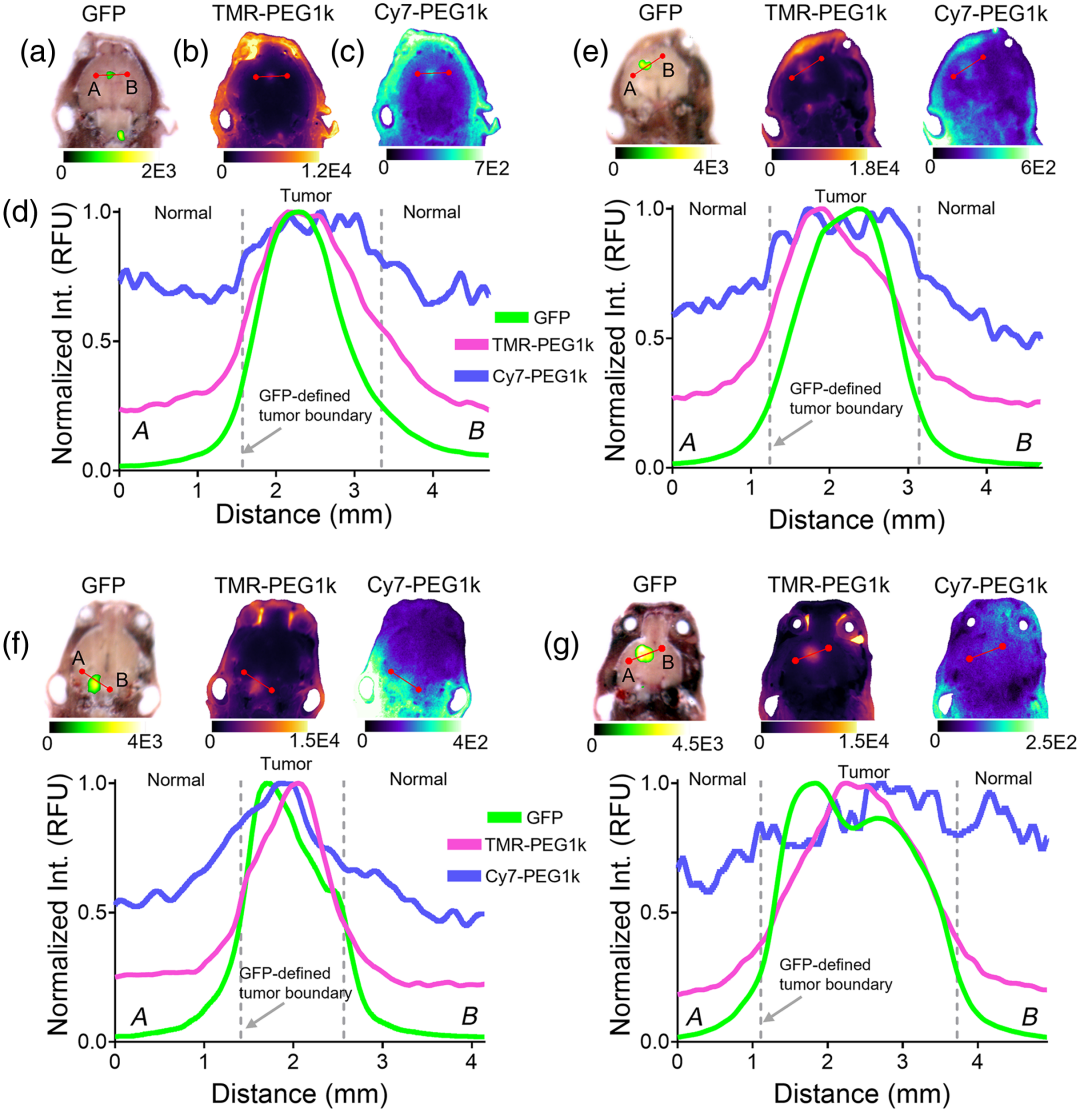
Selected 2D images and line intensity profiles of TMR-PEG1k and Cy7-PEG1k across the tumor region. Sampled 2D fluorescence images of (a) GFP, (b) TMR-PEG1k, and (c) Cy7-PEG1k for a single mouse. (d) Normalized fluorescence intensity profiles of GFP and contrast agent fluorescence associated with the images shown in panels (a)–(c). (e)–(g) Use the same data display structure as panels (a)–(c) for three additional animals.

**Fig. 5:**
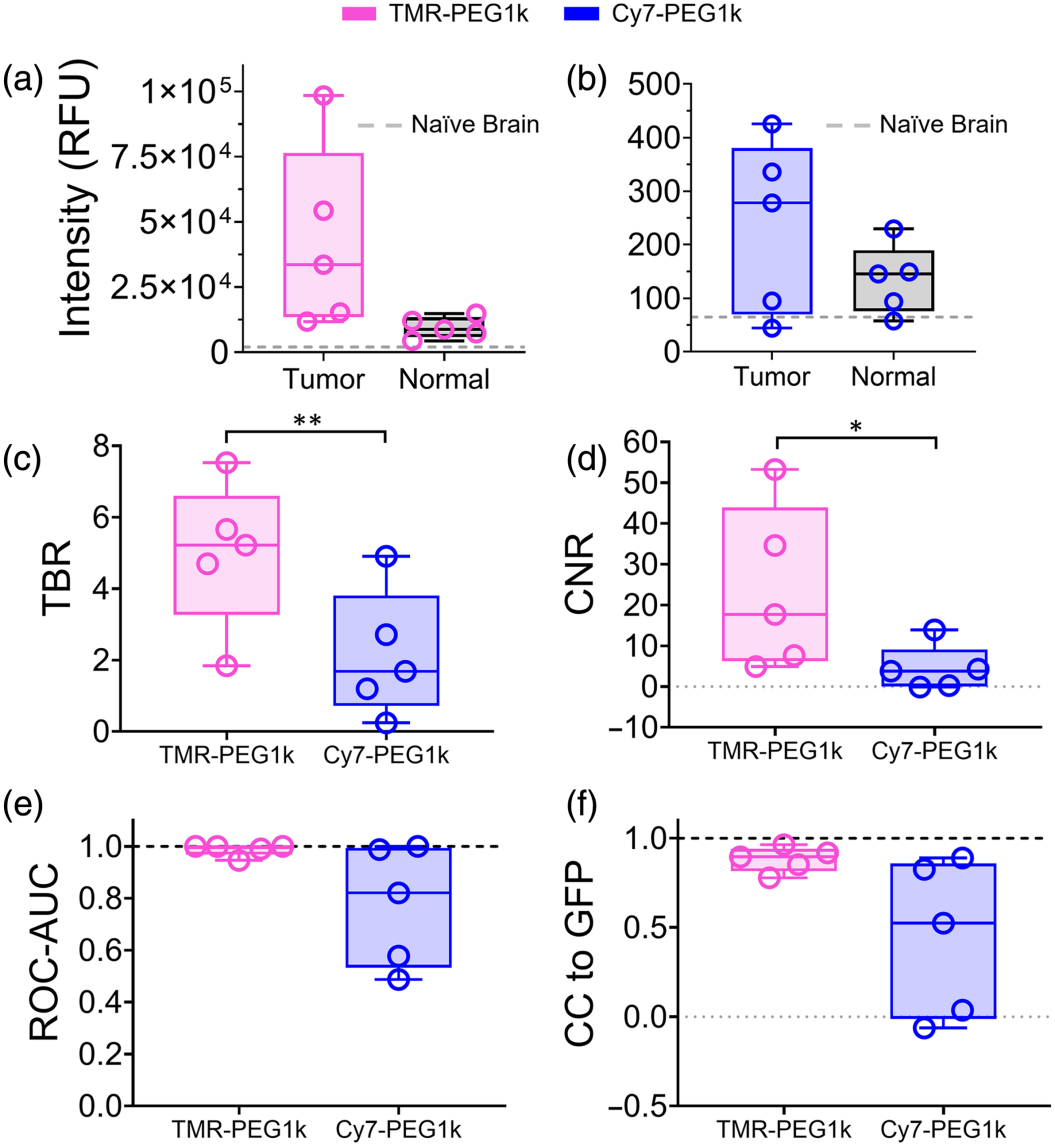
Performance metrics for TMR-PEG1k and Cy7-PEG1k in whole brains. Each dot represents an individual animal, and each plot is represented as a boxplot with a median and interquartile range. Mean intensity in tumor and background brain tissue for TMR-PEG1k (a) and Cy7-PEG1k (b). (c) TBR. (d) CNR. (e) ROC-AUC. (f) Normalized cross-correlation (CC) with GFP fluorescence volume. Note: *p<0.05; **p<0.01.

Examination of selected 2D images further informs the differential uptake of the two agents. Figure 4 shows 2D images of GFP, TMR-PEG1k, and Cy7-PEG1k with corresponding normalized line intensity profiles for four animals. The dotted lines represent the GFP-defined tumor boundary. These results indicate that TMR-PEG1k more closely mimics the GFP fluorescence than Cy7-PEG1k, the latter of which exhibited a lower relative intensity between the GFP-defined tumor and neighboring background region in all cases. This is further confirmed by examining the mean intensity values in both tumor and normal brain for all animals in Fig. 5(a).

Further quantitative analysis of the imaging volumes confirmed the superior performance of TMR-PEG1k compared with the Cy7 version. Figure 5 provides mean intensities in tumor and normal brain, TBR, CNR, ROC-AUC, and CC computed for both agents (using the full volumetric data) and displayed as box plots with median and interquartile ranges. TMR-PEG1k provided a median TBR = 5.2, which was 3.1 times greater than Cy7-PEG1k’s TBR = 1.7 (p=0.0037). TMR-PEG1k also exhibited significantly higher CNR than Cy7-PEG1k (17.7 versus 3.8, respectively, p=0.046).

The ROC-AUC results for these agents are displayed as box plots with a dotted black line representing the highest ROC-AUC of 1 in Fig. 5(e). The median ROC-AUC values were 0.999 and 0.821 for TMR-PEG1k and Cy7-PEG1k, respectively. Notably, the intersubject range of values was small for TMR-PEG1k, with a minimum of 0.946, compared with that observed for Cy7-PEG1k, which exhibited a wide range, including one subject reporting a value <0.6.

The similarity between the fluorescent contrast agents and GFP tumor volume was assessed by calculating the normalized CC between each respective contrast agent fluorescence volume and the corresponding GFP volume [Fig. 5(f)]. TMR-PEG1k exhibited a median CC to GFP of 0.90, and reported values in all animals were above 0.78. Cy-PEG1k showed a much lower correlation to GFP, exhibiting a median CC of 0.52, which covered a range from −0.06 to 0.89.

**Fig. 6:**
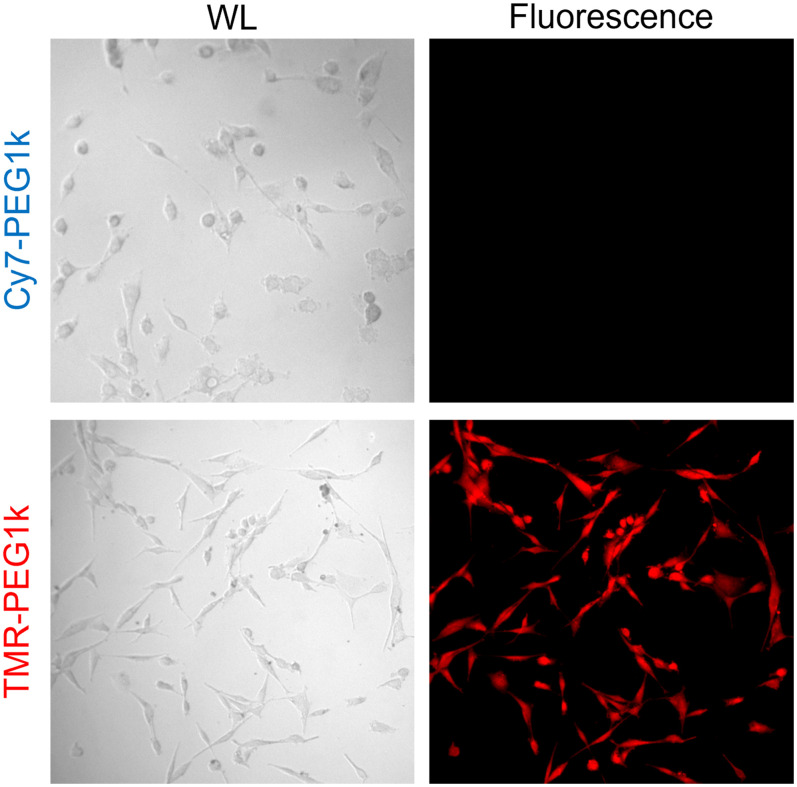
Brightfield and fluorescence microscopy images of U87 cells after incubation with 5  μM of either Cy7-PEG1k or TMR-PEG1k.

To help elucidate the observed behavior *in vivo*, live U87 cells were incubated for 30 min in a 5-μM solution of either agent and then washed with PBS. Fluorescence microscopy images of each agent are shown in Fig. 6, revealing abundant cellular uptake of TMR-PEG1k yet undetectable levels of Cy7-PEG1k. Finally, organ-level biodistributions of the two agents determined from segmented organ structures in the cryotomography volumes are provided in Fig. S4 in the Supplementary Material.

## 4 Discussion and Conclusion

This study determined whether an NIR version of a promising new PEGylated fluorescent contrast agent that currently operates in the visible regime retained the favorable properties of the original. Specifically, we compared the NIR-labeled Cy7-PEG1k to the visible TMR-PEG1k in brain tumor models using high-resolution fluorescence cryotomography. In previous studies, TMR-PEG1k has exhibited highly favorable properties, including very high tumor contrast and diagnostic performance within minutes of administration that persisted for hours and high correlation to contrast-enhanced MRI.^6^ An NIR version with similar behavior could enable highly specific reporting of tumor tissue from subsurface pathologies.

The results reported herein indicate that the Cy7-PEG1k does not retain the properties exhibited by TMR-PEG1k. As compared with the TMR version 30 min after administration, Cy7-PEG1k exhibited lower median TBR (by a factor of 3.1), CNR (by a factor of 4.7), CC to GFP (by a factor of 1.7), and median ROC-AUC decreased by 18%. In addition, although the range of ROC-AUC and CC values was narrow for TMR-PEG1k, they covered a broad range for the Cy7 version of the agent.

Closer inspection of the data reveals that the Cy7 version of the agent was found in normal brain regions and overall exhibited much lower signal intensities than the TMR form. The latter finding is particularly noteworthy because the cryoimaging instrument was shown to be nearly 10 times more sensitive to Cy7-PEG1k than to TMR-PEG1k (Fig. 1). These findings suggest that Cy7-PEG1k either does not accumulate in the tumor as readily or is cleared much faster from the blood than the TMR version, affecting the performance of the compound as a contrast agent. The results of the cell staining study, which showed high uptake of the TMR agent and undetectable retention of the Cy7 version, suggest that cell internalization may be a central contributor to the difference in performance between the two agents.

Physiochemical properties of fluorophores (such as net or surface charge distribution, molecular size, and hydrophobicity) are widely recognized to have a significant impact on *in vivo* behavior of fluorescently labeled molecules.^31^^–^^33^ In this study, the two fluorophores have similar molecular weights and logD values but different charges. The neutral charge of TMR is generally associated with enhanced cell membrane permeability, whereas a positively charged molecule, such as Cy7, often has less propensity to enter cells.^34^ Further studies using NIR fluorophores that more closely match the physicochemical properties of tetramethylrhodamine would serve to elucidate the observed behavior.^35^

The high-resolution, volumetric imaging technique used to assess the performance of these agents is robust and leaves little room for being a source of confounding factors that could explain the stark differences in observed performance between the agents. Notably, the agents were evaluated at the same time post-administration in the same animals with image volumes that were perfectly co-registered to one another and to the GFP channel. Interestingly, the U87-GFP tumor cells implanted locally in the brains of these mice produced multiple tumor regions throughout the brain of every animal. Although Cy7-PEG1k did not perform as hoped in this study, this tumor growth behavior further tested the capabilities of TMR-PEG1k, which continued to show excellent performance even under these more complex pathological brain tissue distributions.

The FGS field in general has shown a preference for developing fluorophores in the NIR, which allows for the detection of fluorescence originating deeper within the tissue. However, many of the fluorophores widely used in the clinic operate in the visible waveband (e.g., fluorescein sodium and protoporphyrin IX), and notable trade-offs exist between the two wavelength regimes. Although visible dyes inform only on superficial structures, they provide much sharper images that are easier to interpret. Conversely, the diffuse nature of NIR fluorescence from deeper structures can confound interpretation in some settings. In this context, the choice of fluorescence regime is likely application-specific, and some may benefit from both when used together. Thus, developing and testing agents across both wavelength regimes is an important pursuit.

The results presented herein indicate that the Cy7-PEG1k tested here is not a suitable NIR version of TMR-PEG1k and is not a compelling candidate for FGS. The change in fluorophore had a dramatic impact on the *in vivo* kinetics, resulting in an agent with poor behavior across performance metrics. However, the evaluation of agents that use alternative NIR fluorophores may exhibit more favorable kinetic profiles. In the meantime, these results also add to the expanding data confirming the high performance of TMR-PEG1k. Advancement of the TMR-PEG1k agent is ongoing, whereas the pursuit of an NIR version will require further consideration.

## Supplementary Material

10.1117/1.JBO.30.12.126004.s01

## Data Availability

All relevant research data is available at (https://doi.org/10.6084/m9.figshare.30629666).
