# Supplementary material for: Evaluation of a near-infrared version of TMR-PEG1k, a high-performance untargeted contrast agent for fluorescence-guided surgery, using fluorescence cryotomography
Source: J Biomed Opt. 2025 Dec 4;30(12):126004. doi: 10.1117/1.JBO.30.12.126004 (PMC12676900; doi:10.1117/1.JBO.30.12.126004)
Supplement: Supplementary file 1 [file JBO_030_126004_SD001.pdf]

Supplementary Material

Title: Evaluation of a near-infrared version of TMR-PEG1k, a high-performance untargeted contrast agent for fluorescence-guided surgery, using fluorescence cryotomography

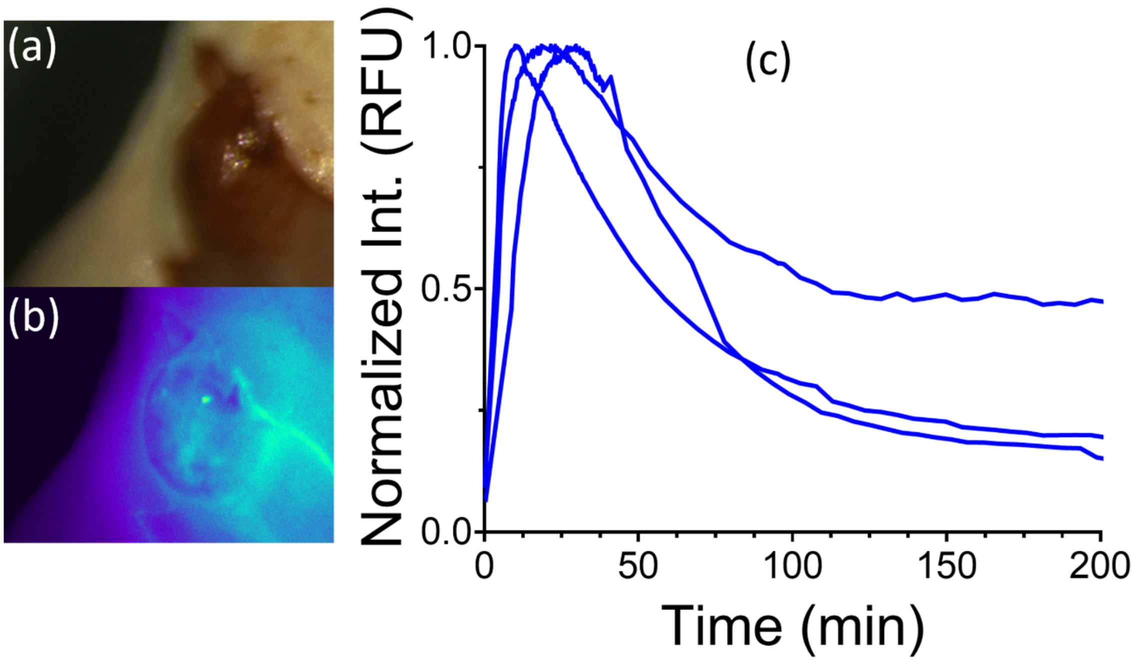

Figure S1. Mean Cy7-PEG1k fluorescence intensity kinetics in exposed tumors: U251 human cells were implanted in the flanks of nude mice. Once tumors reach 1 cm across, animals were anesthetized and the skin incised and moved back to expose the tumors. The tumors were illuminated with a 760 nm excitation laser and imaged with a PCO Edge SCMOS camera with an 800 nm long pass emission filter. Imaging began before administration of Cy7-PEG1k and continued for up to 200 minutes. Example white light RGB and fluorescence images are shown in (a) and (b), respectively. (c) Peak uptake was observed between 10 and 30 minutes amongst three tumors examined (values were baselined to pre-injection images and normalized to the peak value for plotting).

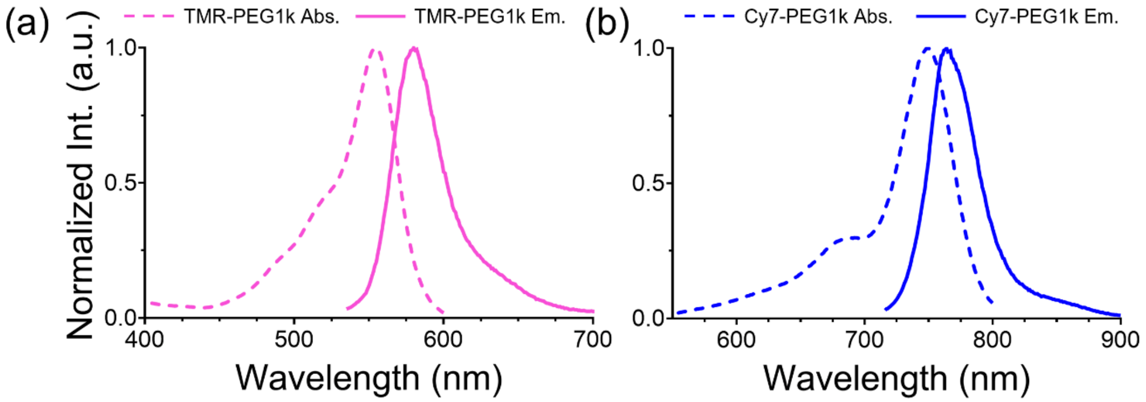

Figure S2. Absorption and emission spectra of TMR-PEG1k (a) and Cy7-PEG1k (b).

	**TMR**	**Cy7**
**Molecular Weight (g/mol)**	**386.45**	**449.66**
**Net Charge**	**0**	**+1**
**LogD**	**4.31**	**4.55**

Figure S3. Physiochemical properties of TMR and Cy7 computed using MarvinSketch (ChemAxon, Budapest, Hungary).

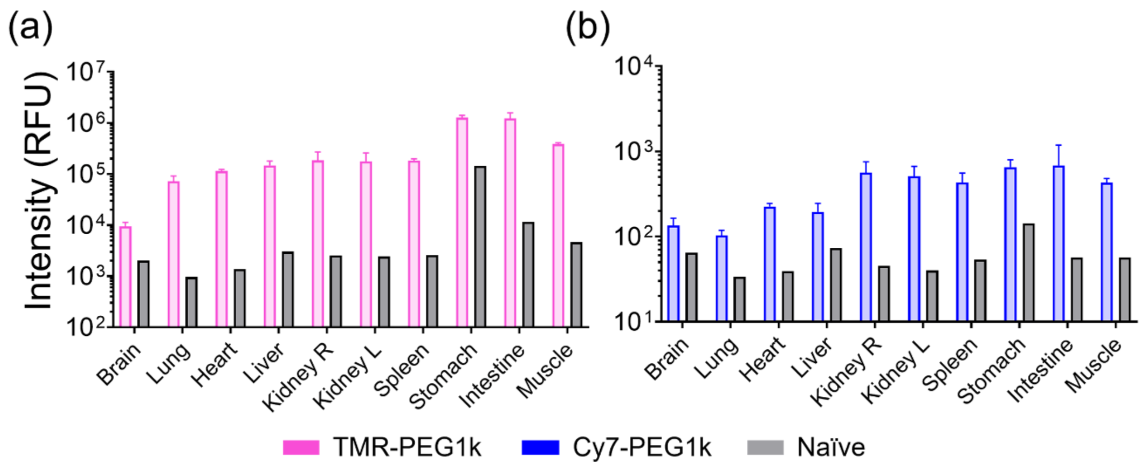

Figure S4. Mean fluorescence intensity values from the TMR-PEG1k and Cy7-PEG1k imaging channels in each organ. These were computed by manually segmenting each organ in each mouse and computing the mean values in those organs.
